# Educating the power: HIV/AIDS and parliamentarians of Pakistan

**DOI:** 10.1186/1478-4505-7-20

**Published:** 2009-09-16

**Authors:** Mohammad A Rai, Alefiyah Rajabali, Muhammad N Khan, Mohammad A Khan, Syed H Ali

**Affiliations:** 1 Aga Khan University, Karachi, Pakistan

## Abstract

Increasing rates of HIV have been recorded amongst the Injection Drug User community from all parts of Pakistan. This has mobilized the health authorities into definitive action before there is a general spread of the epidemic into the Pakistani populace. Lacking any formal research as pertains to HIV policy development in Pakistan, international collaborating agencies, including the United Nations, are aiding in the formulation of a national policy to tackle HIV/AIDS. This article discusses the progress and importance of interventions being conducted amongst the Parliamentarians of Pakistan, relatively unchartered waters. The series of Seminars help to appraise the Parliamentarians of the ground situation as pertains to HIV in their constituencies, aiming to ultimately generate federal and provincial governmental policies, and a solid strategy to combat the spread of HIV/AIDS in Pakistan.

## Introduction

The recent HIV epidemics recorded amongst the Injection Drug User community in various cities of Pakistan have mobilized the health authorities, particularly the international agencies, into realizing HIV as an actual ground reality in this South-East Asian Republic. To prevent a deluge of HIV incidence, various initiatives have been launched. The Pakistani health authorities and their fellow Parliamentarians are being urged to play their part to fight up to this challenge, keeping in mind the dearth of any formal research efforts in Pakistan targeted towards HIV/AIDS policy formulation. The role of prevention as pertains to HIV spread in this country becomes all the more crucial in view of its dire economic and societal indices. Therefore, Pakistani Parliamentarians are being encouraged to understand the dynamics of HIV/AIDS spread in Pakistan through a series of informative seminars, ultimately geared to aid in the formulation of a National HIV/AIDS Policy.

## Discussion

To help Pakistan prepare its HIV/AIDS policy, a diverse preliminary forum was organized two years ago at Islamabad, the capital of Pakistan, in mid-January, 2005. Organized under the umbrella of UNAIDS and Parliamentarians for Global Action (PGA)[1], the seminar served to bring together a worldwide network of over 1300 Parliamentarians from 110 National Parliaments, including legislators from India, Pakistan, Bangladesh, Nepal and Sri Lanka. The objectives were to empower the Parliamentarians to truly understand the extent of the virus in South Asia, outline strategies proven to work in combating the virus, and, most importantly, address the roles Parliamentarians can play to increase political commitment for addressing the epidemic. Both the Pakistani Prime Minister and the UN's Special Envoy were present at this occasion.

In continuation of the Islamabad Declaration, PGA embarked upon a series of HIV sensitization and awareness seminars for Parliamentarians, specially tailored to address the policy needs of each of Pakistan's four provinces. The first Provincial Parliamentary Seminar on HIV, then, took place in Karachi, the capital of the province of Sindh, in January 2006. The fact that the implementation of primary healthcare, education and reproductive health services falls under the authority of provincial legislatures according to the Pakistani constitution, the role of the Provincial Parliamentarians becomes all the more crucial. Various reports of HIV cases in IDUs have emerged from Sindh[2]. The provincial seminar in Sindh provided a rare opportunity for Parliamentarians of rival parties to come together on the HIV/AIDS issue. All four provinces were adequately represented, with special envoys from Sri Lanka and Canada also present. The seminar objectives included enhancing the Parliamentarians' understanding of their own provinces, familiarizing them with priority areas of intervention and outlining a plan of action at both provincial and district levels. Invaluable discussions took place where the people-in-power debated over the formulation of laws and policies against the virus.

The second installment of these awareness campaigns for the Parliamentarians was organized by PGA in December 2006, in Lahore, the capital of the province of Punjab, the most populous province of Pakistan. Apart from serving as a platform for discussion and exchange of ideas on prevention strategies for the spread of the virus, the seminar provided an opportunity for the members of each provincial group to share reviews of action plans previously formulated by each province during the Karachi seminar. This enabled a reassessment and subsequent reinforcement of commitment to the cause.

Spending capital and organizing research garnered towards HIV/AIDS policy development is a scarce commodity in Pakistan. There are no public funds available to channel in this direction, but this should be understandable considering Pakistan's faltering economic and educational standards (the country ranks 77 on the Human Poverty Index[3]). However, all is not bleak, and international organizations including UNAIDS and PGA are making serious efforts to establish and engage research targeted specifically towards policy development.

PGA is making a commendable effort in Pakistan and South Asia to improve HIV awareness on a governmental level. PGA plans to target each of the four Pakistani provinces individually, allowing the Parliamentarians to view and evaluate the HIV policy issues unique to each province. After Sindh and Punjab, the next two provinces in line are Balochistan and the North-West Frontier Province. The plan of action PGA have chalked out for themselves is thorough and systematic. It also, however, comes with a full bag of obstacles and challenges.

Right from its inception, Pakistan, a former British Colony, has been in an out of the vortex of political upheavals - its fate continually tossed between the feudal lords and the military. The consistent absence of true democratic spirit in the country has, consequently, generated a hiatus between the populace and the Parliamentarians.

In the political scenario of Pakistan, the dogma of democracy stems from a very unique perspective. Carefully guarded legacies of family traditions ensure that power is handed down within a select group of individuals. The welfare of the general populace then usually is a circumstance, resultant of the interplay of custom, authority, and tribal wisdom.

Pakistan has come a long way since its first reported HIV case in 1987[4]. UNAIDS latest figures estimate the number of cases bordering 96 thousand[3]. Underreporting and limited surveillance means that the actual number of infected is much higher. The social and health demographics of the county are also extremely distressing, with a low literacy rate (UNESCO estimates female adult literacy to border around 40% in 2006[3]) combined with a burgeoning population growth rate. All in all, the nation already has enough problems on its hands before letting the scourge of HIV jump in as well.

In the recent few years, there have been incidences recorded of HIV outbreaks from all major cities of Pakistan, including Lahore, Karachi and Hyderabad [5,6]. This rapid spread of HIV has certainly shaken the earlier otherwise dormant status-quo in this predominantly Muslim nation, which had been relatively safe from a steep rise in HIV infections for almost two decades, in contrast to its neighbor, India.

The patterns of HIV transmission in Pakistan are similar in many ways to other parts of Asia. The virus traverses the barrier from high-risk communities to the mainstream population. Once this transmission jump has occurred, the increase in HIV incidence is swift and uncontrollable. Along a similar pattern, India experienced an explosive spread of HIV/AIDS in the early 90s. Ironically, the same HIV risk communities, mainly Injection Drug Users (IDUs), that fueled India's epidemic[7], are showing a steady surge of HIV prevalence in Pakistan as well.

In Pakistan, any hope of winning the battle against HIV lies on the prevention front. The relatively low prevalence of HIV infected individuals presents a window of opportunity for the implementation of preventive strategies. Severe lack of resources and expertise in treating the disease threatens to spread the virus into the mainstream population. Once the transmission jump occurs, Pakistan will be fighting a lost battle. The only promise of initiating a change is offered through the involvement of the people who have the power to *bring *the change - the Parliamentarians of Pakistan. Ironically, the HIV initiatives from PGA are the first examples of rally points for all Parliamentarians, regardless of party or demographic advocacy, a rarity in the tormented Pakistani political setup. More effort needs to be made in the same direction. Pakistani politicians must realize that issues such as HIV/AIDS require them to come together with a concerted sincerity that rises above the mandate of their individual political affiliations, and considers the issues in a national and global perspective.

## Conclusion

The HIV situation in Pakistan has not exploded yet[8]. HIV might still be a distant, albeit a very real threat, as evidenced by the spate of epidemics occurring amongst the IDU populace of Pakistan[9]. Steps taken right now will go a long way in battling HIV/AIDS. But in order to bring and sustain change on the ground, the government needs to extend its full support and participation. The role of the Pakistani Parliamentarians becomes extremely important, as they are the vehicles that can bring about a change, not just in initiating various preventive programs directed under the auspices of a National HIV/AIDS Policy, but also helping to remove stigma associated with the disease, all aiding greatly to prevent a steep incline in HIV/AIDS cases.

## Competing interests

The authors declare that they have no competing interests.

## Authors' contributions

All authors were part of the organizing committee for the first Provincial Parliamentary Seminar on HIV which took place in Karachi. MAR, AR and SHA were involved in drafting this manuscript.
